# Follow-up PET/CT of alveolar echinococcosis: Comparison of metabolic activity and immunodiagnostic testing

**DOI:** 10.1371/journal.pone.0270695

**Published:** 2022-06-29

**Authors:** Lars Husmann, Ansgar Deibel, Stephan Skawran, Bruno Ledergerber, Urs J. Muehlematter, Barbara Hasse, Martin W. Huellner, Caecilia S. Reiner, Beat Muellhaupt

**Affiliations:** 1 Department of Nuclear Medicine, University Hospital Zurich, University of Zurich, Zurich, Switzerland; 2 Division of Gastroenterology and Hepatology, University Hospital Zurich, University of Zurich, Zurich, Switzerland; 3 Division of Infectious Diseases and Hospital Epidemiology, University Hospital Zurich, University of Zurich, Zurich, Switzerland; 4 Institute of Diagnostic and Interventional Radiology, University Hospital Zurich, University of Zurich, Zurich, Switzerland; Spedali Civili of Brescia, University of Brescia, ITALY

## Abstract

**Purpose:**

To investigate the potential role of follow-up ^18^F-fluorodeoxyglucose (FDG) positron emission tomography/computed tomography (PET/CT) in therapy control of inoperable patients with alveolar echinococcosis.

**Materials and methods:**

In this single-center retrospective cohort study, 48 PET/CT of 16 patients with confirmed alveolar echinococcosis were analysed. FDG-uptake of the most active echinococcosis manifestation was measured (i.e., maximum standardized uptake value (SUVmax) and in relation to background activity in normal liver tissue (SUVratio)) and compared to immunodiagnostic testing. For clinical patient follow-up, patient demographics, laboratory data, including *E*. *granulosus* hydatid fluid (EgHF) antibody units (AU) as well as clinical and treatment information were assessed for all patients at the time of PET/CT, and at the last recorded clinical visit.

**Results:**

Metabolic activity of PET/CT measured in the echinococcosis manifestation was significantly correlated with EgHF AU (p < 0.001). The differences in metabolic activity of echinococcosis manifestations between two consecutive PET/CT examinations of the same patient and differences in EgHF AU in the respective time intervals displayed a significant positive correlation (p = 0.01). A trend for a more rapid decline in SUVratio liver over time was found in patients who stopped benzimidazole therapy versus patients who did not stop therapy (p = 0.059).

**Conclusion:**

In inoperable patients with alveolar echinococcosis, the course of metabolic activity in follow-up PET/CT is associated to the course EgHF antibody levels. Both parameters may potentially be used to evaluate the course of the disease and potentially predict the duration of benzimidazole therapy.

## Introduction

Alveolar echinococcosis is a rare parasitic disease, most frequently affecting the liver as a slow-growing tumor-like lesion. In the initial phase of the disease, alveolar echinococcosis often remains asymptomatic, and diagnosis is often made many years after infection and at inoperable stages. Anthelmintic treatment with benzimidazole has led to a major improvement in survival [1–5] in patients with inoperable alveolar echinococcosis. The therapy is considered parasitostatic [6], and lifelong medication is recommended. However, several reports have shown, that occasionally treatment effects may be parasitocidal, and treatment may be discontinued without risk of recurrence in some cases [7, 8]. Immunodiagnostic testing and ^18^F-fluorodeoxyglucose (FDG) positron emission tomography/computed tomography (PET/CT) are used to determine such cases, i.e., in patients with negative serology and no residually increased metabolic activity in PET/CT, therapy may be discontinued [6, 8]. As benzimidazole therapy is occasionally associated with increased morbidity, the prediction of therapy duration would be desirable, especially in cases with therapy side effects. To date, *E*. *multilocularis*-specific serological tests and imaging (usually with ultrasound or magnetic resonance imaging) are used to monitor the course of alveolar echinococcosis, but these methods are not apt to predict therapy duration.

We hypothesized, that the metabolic activity of echinococcus manifestations in follow-up PET/CT may add additional information on the course of alveolar echinococcosis. Therefore, the aim of our study was to compare serology and quantitative measurements of sequential PET/CT scans in monitoring benzimidazole therapy in patients with inoperable alveolar echinococcosis.

## Materials and methods

### Study design and data collection

For this retrospective study, we screened patient imaging reports of the years 2005 to 2019 for the term “echinococcus” and selected those patients who underwent a PET/CT scan during this time. We included consecutive patients who had an inoperable infection with *E*. *multilocularis*, underwent PET/CT at our institution between the years 2005 and 2019 and had at least one additional follow-up PET/CT scan until the bginning of the year 2022.

The local ethics committee approved the study, namely the Kantonale Ethikkomission Zürich (BASEC-Nr. 2018–01855). All patients examined between 2016 and 2019 gave written informed consent to the retrospective use of their clinical data for research. For patients scanned between the years 2005 and 2015, informed consent was waived by the local ethics committee, namely the Kantonale Ethikkomission Zürich (BASEC-Nr. 2018–01855). All procedures were performed in accordance with the 1964 Helsinki declaration and its later amendments.

### Imaging data acquisition and image analysis

All PET/CT examinations were performed at our institution and followed the same basic study protocols. Patients fasted for at least four hours, intravenous injection of FDG was body weight adjusted, the standardized uptake time was 60 minutes in supine position, a non contrast-enhanced CT scan was performed and used for attenuation correction, and imaging was acquired with arms overhead whenever possible. Body weight, height, and blood glucose level were measured prior to imaging (blood glucose levels <12 mmol/l were accepted [9]). During the study period, five different types of PET/CT scanners were used at our institution, i.e. Discovery 690, Discovery 710, Discovery ST16, Discovery VCT, and Discovery MI (all GE Healthcare, Waukesha, WI). PET/CT data sets were retrospectively reanalysed in consensus by two experienced and doubly board certified radiologists and nuclear medicine physicians on a dedicated workstation (Advantage Workstation, Version 4.6; GE Healthcare Biosciences, Pittsburgh, PA). Readers were blinded to all clinical patient data. To compensate for differences in sensitivity of the different PET/CT scanner generations, we measured the metabolic activity (maximum standardized uptake values (SUVmax)) in the echinococcus manifestation with the highest uptake, as well as the uptake in normal/non-infected liver tissue and in the mediastinal blood pool, and calculated SUVratios, i.e.: SUVratio liver (SUVmax of the echinococcus manifestation divided by the SUVmax of normal/non-infected liver tissue) and SUVratio blood (SUVmax of the echinococcus manifestation divided by the SUVmax of the mediastinal blood pool).

### Immunodiagnostic testing

All enzyme-linked immunosorbent assays (ELISA) for *E*. *granulosus* hydatid fluid (EgHF) were carried out as previously described in detail by Schweiger et al. [10].

### Patient follow-up

For clinical patient follow-up, electronic patient charts of all patients were reviewed (last follow-up in February 2022). Patient demographics, laboratory data including EgHF—ELISA antibody units (AU) as well as clinical and treatment information were assessed for all patients at the time of PET/CT, and at the last recorded clinical visit.

### Statistical analyses

Variables were expressed as median and IQR (25th, 75th percentiles) or percentages. Spearman rank-correlations were calculated to compare absolute values of SUVratio and EgHF AU as well as the differences (delta) of both variables between two PET/CT examinations in the same patient and the corresponding antibody values. Furthermore, mixed-effects multi level regression was used to analyse the decline in SUVratio and EgHF AU over time, to determine whether patients with a steeper decline were more likely to stop benzimidazole therapy. Finally, we compared differences between delta SUVratio and delta EgHF antibodies in patients with and without benzimidazole therapy using Wilcoxon signed-rank test. A two-tailed p-value of <0.05 was considered to indicate statistical significance. Statistical analyses were performed using commercially available software (Stata/SE, Version 17.0, StataCorp, College Station, TX).

## Results

### Patient population

Our data bank search yielded 219 patients with 389 PET/CT examinations, in which the term “echinococcus” was mentioned in any written imaging report and a PET/CT was performed between the years 2005 and 2019. Two-hundred-and-three of these patients were not included in the final study population due to the following reasons: no clinical confirmation of alveolar echinococcosis (n = 120, 55%), no follow-up PET/CT available (n = 86, 39%), EgHF antibodies at baseline PET/CT equal to zero (n = 11, 5%), and operative resection of echinococcosis between the initial and the first follow-up PET/CT scan (n = 2, 1%).

Hence, 48 PET/CT were performed in 16 patients with serology-confirmed alveolar echinococcosis (six patients had two PET/CT scans, five had three, four had four and one patient had five PET/CT scans). Demographics of the final patient population are displayed in Table 1.

**Table 1:** Patient demographics.

Number of patients	16
Number of PET/CT examinations	48
Female gender, n (%)	8 (50%)
Median age in years (IQR)	54 (42–85)
Median age in years at initial PET/CT (IQR)	51 (40–83)
Median age in years at follow-up PET/CT (IQR)	56 (42–85)
Median weight in kg (IQR)	63 (54–105)
Median weight at initial scan in kg (IQR)Number of curative operations, n (%)	59 (45–102)
Median weight at follow-up scan in kg (IQR)Number of curative operations, n (%)	67 (57–105)

### PET/CT

**Fig 1:**
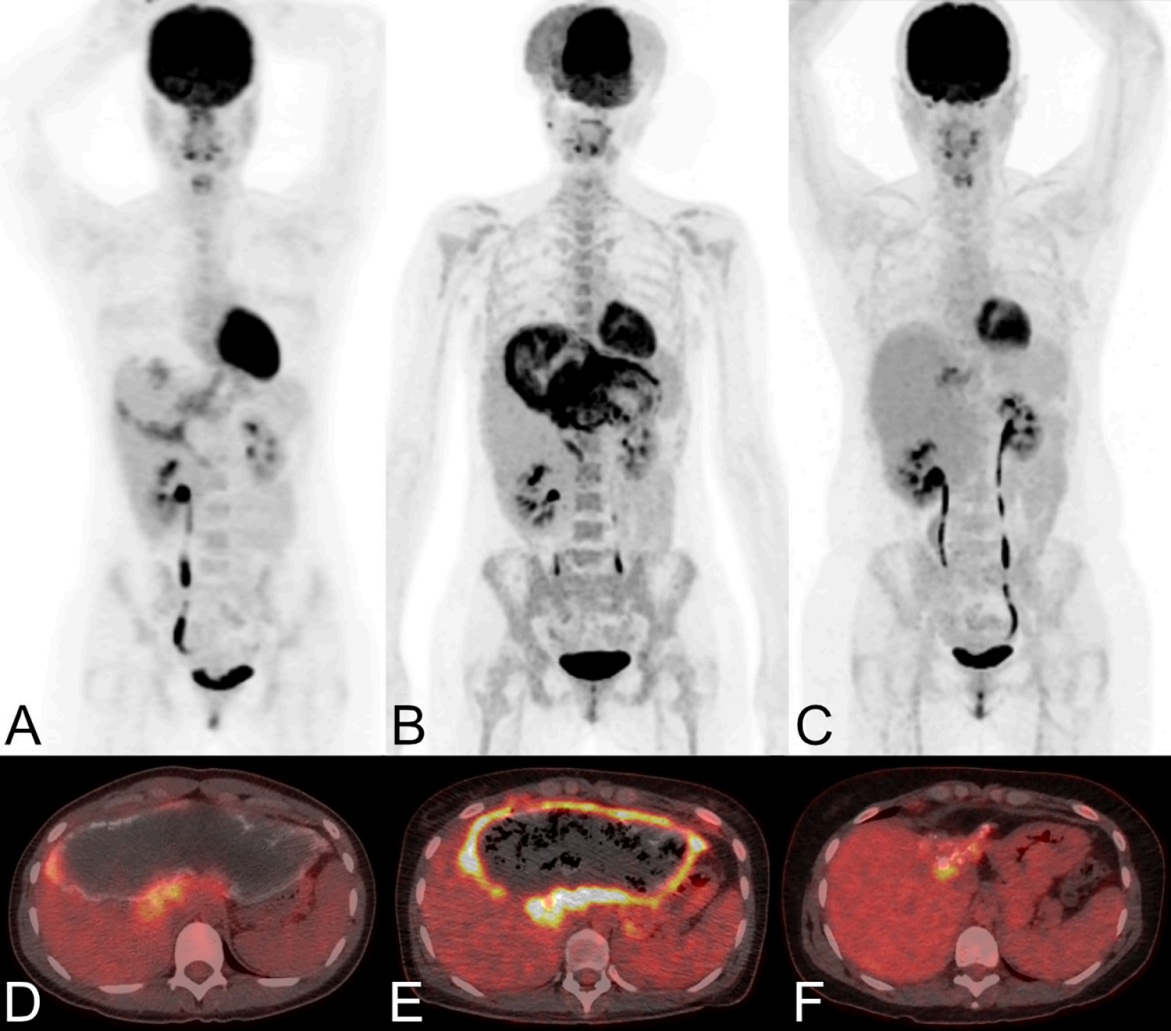
Three PET/CT scans performed in a female patient with a large hepatic manifestation of alveolar echinococcosis. The patient (patient 04 in **Table 2**) was 24 years old in 2005 (maximum intensity reconstructions of PET (A) and fused PET/CT images (D)), 32 years old in 2012 (B and E), and 40 years old in 2020 (C and F). She was treated with albendazol since 1997 and therapy was ongoing at the last clinical follow-up in 2021. In 2005, PET/CT showed moderate metabolic activity (A and D), EgHF AU were 58. In 2012, the patient presented with fever and was diagnosed with cholangitis and subsequently treated with antibiotics—PET/CT showed intense metabolic activity in the periphery of the echinococcosis manifestation (B and E), EgHF AU were lower than in 2005 (i.e., 46). In 2020 (C and F), PET/CT again showed moderate metabolic activity to a lesser extent, EgHF AU were 13. The large discrepancy in trends in this case, may indicate, that metabolic activity and EgHF antibody levels may have to be evaluated independently, when treating patients with alveolar echinococcosis.

**Fig 2:**
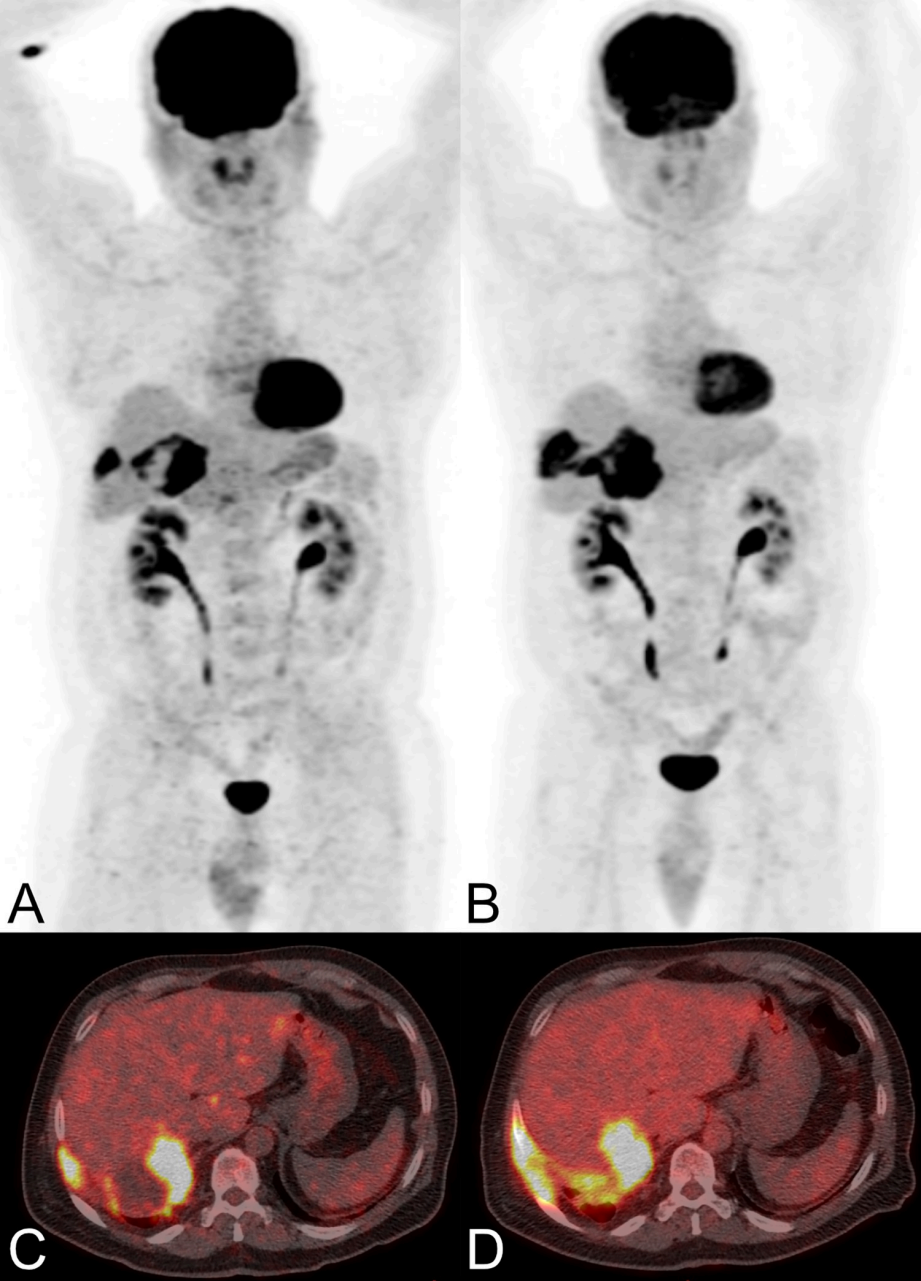
Two PET/CT scans performed in a male patient with a hepatic manifestation of alveolar echinococcosis involving the chest wall. The patient (patient 09 in **Table 2**) was 55 years old in 2008 (maximum intensity reconstructions of PET (A) and fused PET/CT images (C)), and 58 years old in 2011 (B and D) and was treated with mebendazole from 2001 to 2011. The follow-up PET/CT 2011 (B and D) documented progression of disease with increasing involvement of the chest wall as compared to the PET/CT from 2008 (A and C) (EgHF AU increased from 34 to 35, SUVratio liver was continuously high at 4.6), and therapy was subsequently changed to albendazole. The patient died in 2017 of unknown cause.

PET/CT was acquired after intravenous injection of FDG (i.e., 326 Megabecquerel (interquartile range (IQR) 283–400) (Figs 1 and 2). Median SUVmax of the echinococcosis manifestations with the highest FDG-uptake (located in the liver in 14 patients, in the lung in one patient, and in the peritoneum in one patient) was 3.7 (2.8–18.5); median background measurements were 3.0 (2.5–4.6) in non-infected liver tissue and 2.3 (1.9–4.0) in the mediastinal blood pool.

**Fig 3:**
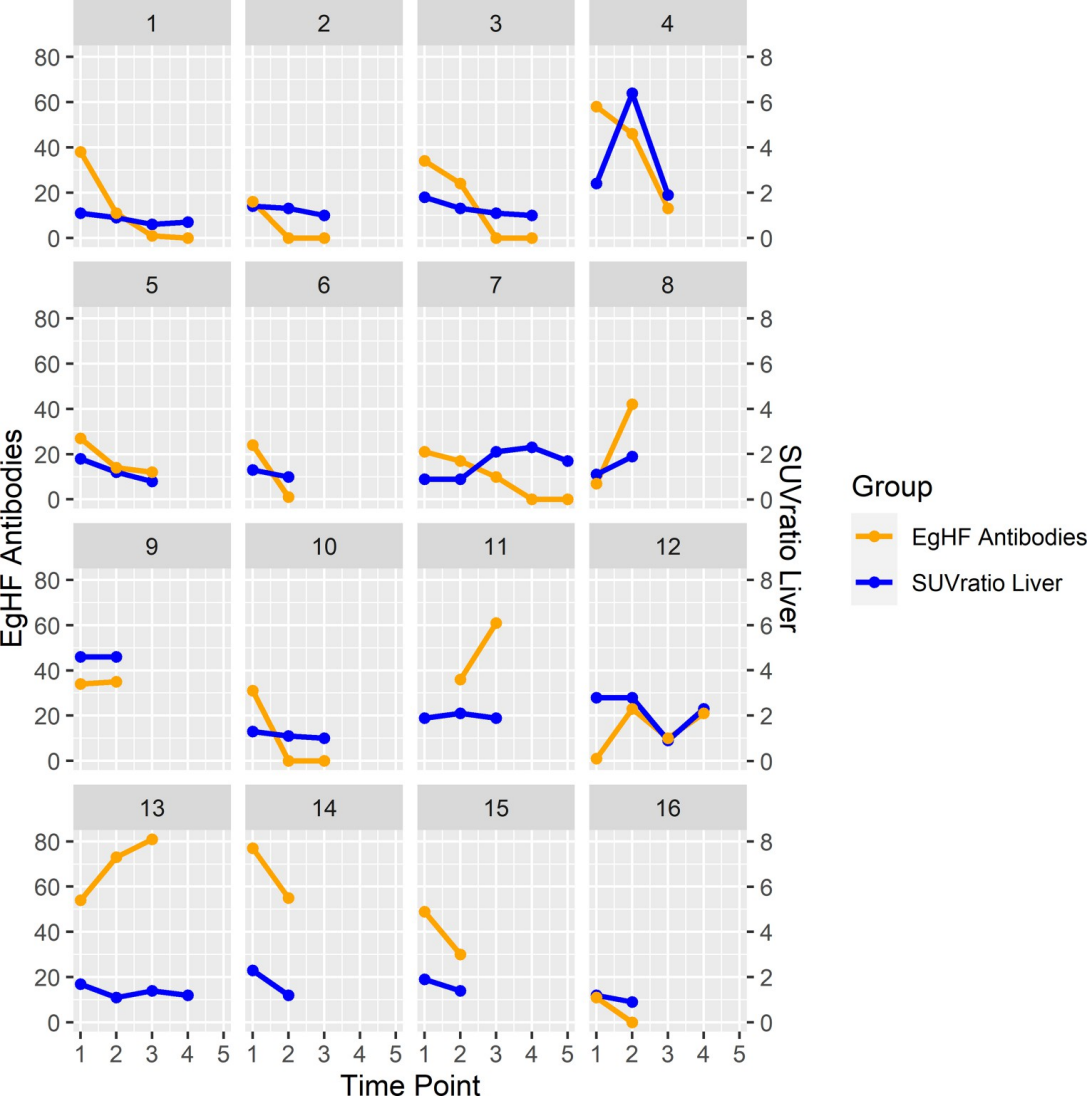
The graphs display the courses of EgHF antibody units and SUVratio liver over time in all 16 included patients with alveolar echinococcosis.

The median SUVratio of echinococcosis manifestations and non-infected liver tissue (i.e., SUVratio liver) was 1.3 (1,0–6.4), and the median SUVratio of echinococcosis manifestations and mediastinal blood pool (i.e., SUVratio blood) was 1.8 (1.3–7.6); individual values are displayed in Table 2 and Fig 3. The median difference of SUVratio liver in echinococcosis manifestations between two PET/CT examination in the same patient (i.e., delta) was -0.2 (0.1 to -4.6), and -0.2 (0.1 to -5.3) for SUVratio blood. In patients on continuous benzimidazole therapy between two PET/CT examinations (23 delta values in 12 patients) the median delta SUVratio liver was -0.2 (0.1 to -4.6), while it was -0.3 (0.0 to -1,1) in patients without continuous treatment (i.e., either no treatment at both time points, or no treatment at one of the time points (9 delta values in 8 patients)). Respective values for delta SUVratio blood were -0.2 (0.1 to -5.3), and -0.1 (-0.1 to -1.0), respectively; differences were not statistically significant.

**Table 2:** Quantitative PET/CT and outcome data of all included patients with inoperable alveolar echinococcosis.

Pat	Age^1^	PET/CT 1			PET/CT 2			PET/CT 3			PET/CT 4			PET/CT 5			Latest clinical follow-up			
		SUV ratio liver	SUV ratio blood	AB EgHF	SUV ratio liver	SUV ratio blood	AB EgHF	SUV ratio liver	SUV ratio blood	AB EgHF	SUV ratio liver	SUV ratio blood	AB EgHF	SUV ratio liver	SUV ratio blood	AB EgHF	Alive	End of treatment	Years after last PET/CT	Years after end of treatment
01	48	1.1	1.2	38	***0*.*9***	***1*.*2***	***11***	***0*.*6***	***1*.*0***	***1***	***0*.*7***	***1*.*0***	***0***	n.a.	n.a.	n.a.	yes	no	10	n.a.
02	44	***1*.*4***	***1*.*8***	***16***	***1*.*3***	***1*.*7***	***0***	***1*.*0***	***1*.*3***	***0***	n.a.	n.a.	n.a.	n.a.	n.a.	n.a.	yes	no	14	n.a.
03	38	***1*.*8***	***2*.*2***	***34***	***1*.*3***	***2*.*0***	***24***	***1*.*1***	***1*.*6***	***0***	***1*.*0***	***1*.*0***	***0***	n.a.	n.a.	n.a.	yes	yes	13	6
04	24	***2*.*4***	***3*.*2***	***58***	***6*.*4***	***7*.*6***	***46***	***1*.*9***	***2*.*3***	***13***	n.a.	n.a.	n.a.	n.a.	n.a.	n.a.	yes	no	16	n.a.
05	51	***1*.*8***	***2*.*3***	***27***	***1*.*2***	***1*.*6***	***14***	***0*.*8***	***1*.*2***	***12***	n.a.	n.a.	n.a.	n.a.	n.a.	n.a.	yes	yes	13	6
06	63	***1*.*3***	***1*.*6***	***24***	1.0	1.5	1	n.a.	n.a.	n.a.	n.a.	n.a.	n.a.	n.a.	n.a.	n.a.	no^2^	yes	1	1
07	65	***0*.*9***	***1*.*3***	***21***	0.9	1.4	17	***2*.*1***	***3*.*5***	***10***	***2*.*3***	***2*.*7***	***0***	***1*.*7***	***1*.*9***	***0***	no^3^	no	13	n.a.
08	83	1.1	1.6	7	1.9	1.6	42	n.a.	n.a.	n.a.	n.a.	n.a.	n.a.	n.a.	n.a.	n.a.	yes	yes	5	9
09	55	***4*.*6***	***6*.*6***	***34***	***4*.*6***	***5*.*8***	***35***	n.a.	n.a.	n.a.	n.a.	n.a.	n.a.	n.a.	n.a.	n.a.	no^3^	no	8	n.a.
10	25	1.3	1.8	31	***1*.*1***	***1*.*3***	***0***	***1*.*0***	***1*.*3***	***0***	n.a.	n.a.	n.a.	n.a.	n.a.	n.a.	no^4^	no	12	7 ^4^^,^^5^
11	50	***1*.*9***	***2*.*3***	***n*.*a*.**	***2*.*1***	***2*.*5***	***36***	***1*.*9***	***1*.*9***	***61***	n.a.	n.a.	n.a.	n.a.	n.a.	n.a.	yes	no	12	n.a.
12	40	***2*.*8***	***3*.*9***	***1***	***2*.*8***	***2*.*9***	***23***	***0*.*9***	***0*.*9***	***10***	***2*.*3***	***3*.*4***	***21***	n.a.	n.a.	n.a.	yes	no	9	n.a.
13	62	1.7	2.5	54	***1*.*1***	***1*.*7***	***73***	***1*.*4***	***1*.*8***	***81***	***1*.*2***	***2*.*1***	n.a.	n.a.	n.a.	n.a.	no^3^	no	2	n.a.
14	40	2.3	2.8	77	***1*.*2***	***1*.*9***	***55***	n.a.	n.a.	n.a.	n.a.	n.a.	n.a.	n.a.	n.a.	n.a.	yes	no	3	n.a.
15	63	***1*.*9***	***2*.*8***	***49***	***1*.*4***	***2*.*6***	***30***	n.a.	n.a.	n.a.	n.a.	n.a.	n.a.	n.a.	n.a.	n.a.	yes	no	4	n.a.
16	63	1.2	1.6	11	***0*.*9***	***1*.*3***	***0***	n.a.	n.a.	n.a.	n.a.	n.a.	n.a.	n.a.	n.a.	n.a.	yes	yes	5	4

***Bold*** numbers indicate ongoing benzimidazole therapy at the time of examination. PET/CT: positron emission tomography/computed tomography; SUV: standardized uptake value; n.a.: not applicable; AB: antibody

^1^ age at initial PET/CT

^2^ due to pancreatic cancer

^3^ cause of death unknown

^4^ due to glioblastoma

^5^ restart of albendazol treatment after a six year pause.

### EgHF antibodies

Median EgHF AU were 19.0 (1.0–81.0). The median difference between measurements at two time points corresponding to two PET/CT examinations in one patient (i.e., delta) was -7.0 (0.5 to -33.0); individual values are displayed in Table 2 and Fig 3. In repetitive measurements in patients on continuous benzimidazole therapy (22 delta values in 12 patients), the median delta of EgHF AU was -1,5 (0.8 to -33), while it was -15.0 (-4.0 to -31.0) for patients without continuous treatment (9 delta values in 8 patients), differences were not statistically significant.

### Relation of quantitative PET/CT measurements and EgHF antibodies

**Fig 4:**
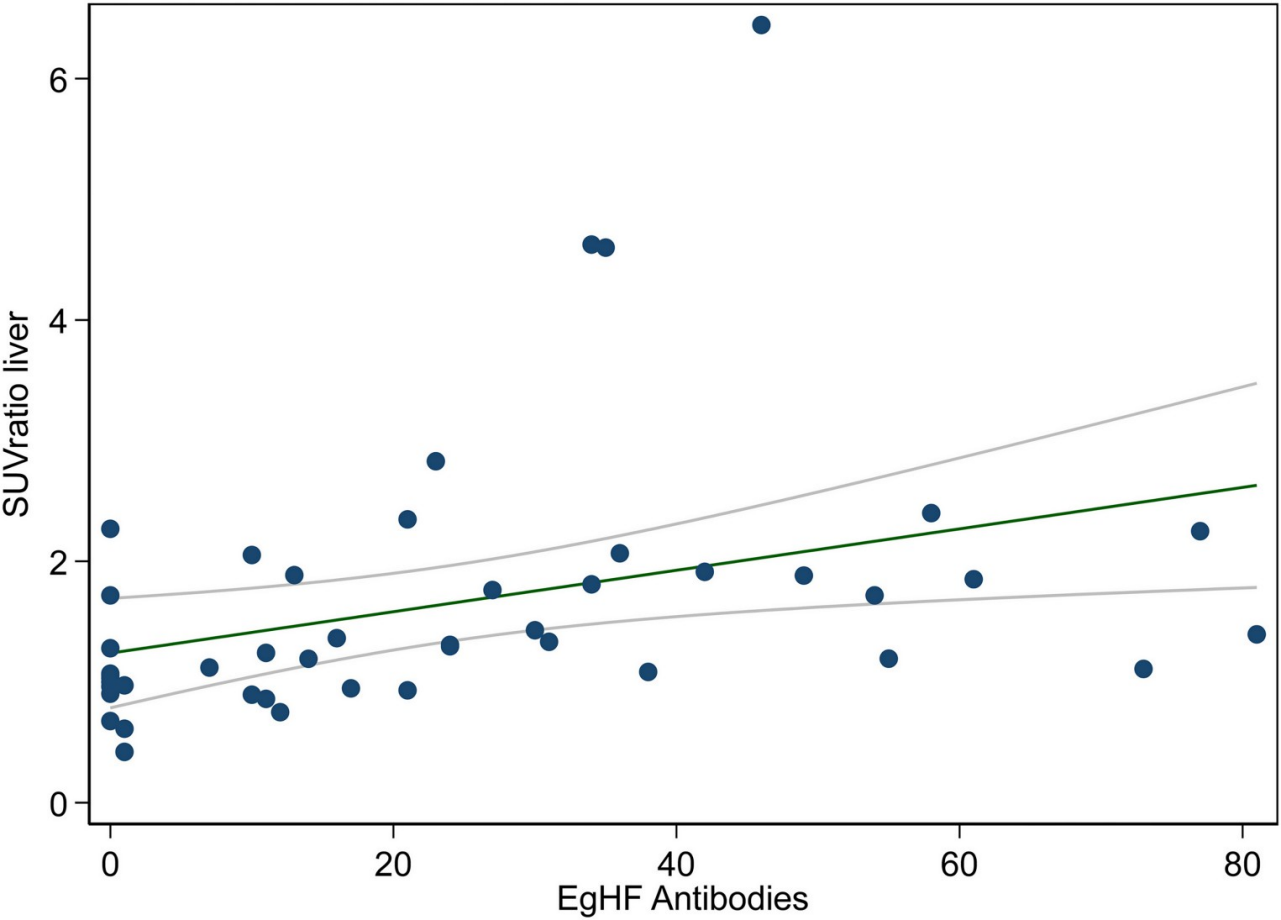
**The graph displays linear regression (green line) and 95% confidence intervals (grey curves) of the SUVratio liver (highest metabolic activity of an echinococcosis manifestation divided by metabolic background activity in normal liver tissue) and EgHF antibody units.** A significant correlation of both variables was found (p < 0.001).

**Fig 5:**
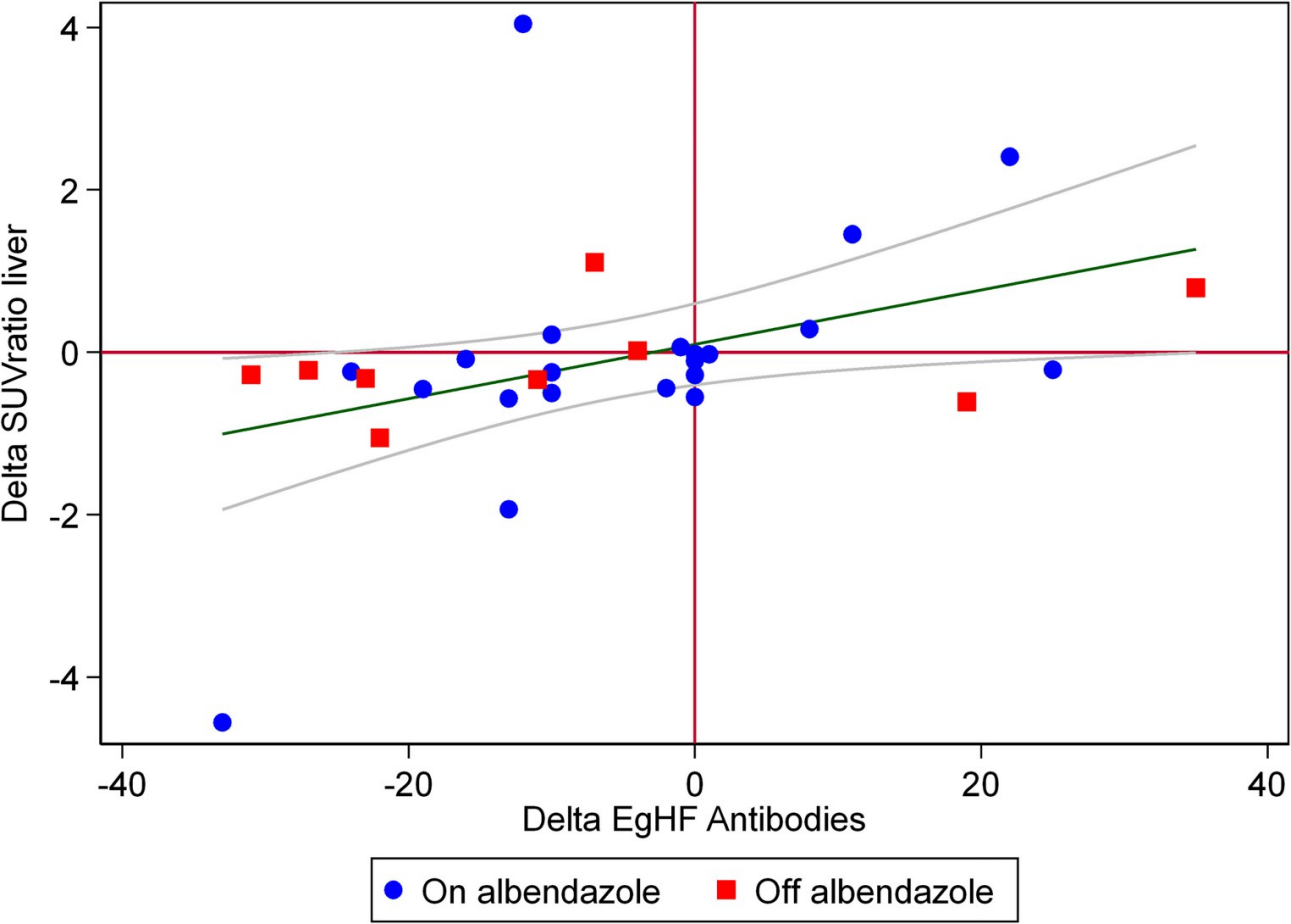
**The graph displays linear regression (green line) and 95% confidence intervals (grey curves) of the difference (i.e., delta) of SUVratio liver in echinococcosis manifestations between two PET/CT examination of the same patient and the delta of EgHF antibodies in the respective time intervals.** Overall, a significant correlation of both variables was found (p = 0.01). When comparing, patients on continuous benzimidazole therapy (blue dots) to patients without continuous treatment (red squares), no significant difference was determined.

Metabolic activity in PET/CT measured in the echinococcosis manifestation was significantly correlated with EgHF AU (p < 0.001 for SUVratio liver and p < 0.001 for SUVratio blood) (Fig 4). Furthermore, the difference (i.e., delta) of SUVratio liver in echinococcosis manifestations between two PET/CT examination of the same patient and the delta of EgHF antibody levels in the respective time intervals displayed a significant correlation (p = 0.01) (Fig 5). When comparing patients on continuous benzimidazole therapy to patients without continuous treatment, no significant difference was found. We found a trend for a more rapid decline in log10 transformed SUVratio liver over time in patients who stopped benzimidazole therapy versus patients who did not stop therapy (0.021 vs. -0.002, p = 0.059). Likewise, EgHF AU had a steeper decline in patients who stopped benzimidazole therapy versus patient who did not stop therapy (3.12 vs. 1.56 units), but differences were not statistically significant (p = 0.15).

Noteably, despite the overall correlation of the metabolic activity in PET/CT and EgHF antibody levels, in some patients large discrepancies between trends were documented (e.g., patient 4 in Figs 1 and 3, and Table 2).

### Outcome

Patients were clinically followed for a median of 12.1 years (IQR 5.8–30.8) after their initial diagnosis and for a median of 3.9 years (IQR 0.9–8.6) after their last follow-up PET/CT examination. In six of 16 patients (38%), benzimidazole therapy was successfully stopped after 4.7 (IQR 2.2–14.9) years (in one of these patients benzimidazole therapy was reinitiated after a six-year pause due to a new diagnosis of glioblastoma (patient 10 in Table 2)). At the time of the first PET/CT examination, benzimidazole therapy was already started in 10 (63%) patients. Of 32 follow-up examinations, 29 (91%) were performed with ongoing benzimidazole therapy (two patients were treated with mebendazole, all others with albendazole) (Table 2).

Five patients (38%) died during follow-up. The cause of death was unknown in three patients, one died due to pancreatic cancer, and one due to a glioblastoma (Table 2).

## Discussion

We compared *E*. *multilocularis*-specific serology and quantitative measurements of PET/CT in monitoring benzimidazole therapy in patients with inoperable alveolar echinococcosis. Our study results are: (i) Metabolic activity of the echinococcosis manifestation correlated with EgHF antibody levels. (ii) The difference over time in metabolic activity of echinococcosis manifestations between two PET/CT examination of the same patient and the difference in EgHF antibody levels in the respective time intervals had a significant correlation. (iii) A trend for a more rapid decline in SUVratio liver over time was found in patients who stopped benzimidazole therapy compared to patients who did not stop therapy (p = 0.059).

EgHF ELISA is used for primary immunodiagnosis of alveolar echinococcosis, and offers a high diagnostic sensitivity of 95% [11] in clinical routine. For follow-up, other antibodies (i.e., EM2-plus or Em18 ELISA) are established in clinical routine, exhibiting high performance in detecting disease recurrence after surgical resection of alveolar echinococcosis. However, antibody tests are less reliable in inoperable patients and in patients after incomplete resection [12]. Furthermore, EM2-plus or Em18 ELISA are used to determine the viability of alveolar echinococcosis [13, 14] and hence guide treatment decisions during the course of incompletely resected or inoperable disease [15]. They, however, seem insufficiently discriminant to substantiate a decision on treatment withdrawal [15, 16]. EgHF—ELISA is also routinely performed in many institutions in follow-up of alveolar echinococcosis, however, its clinical value remains unclear.

EgHF has previously been used successfully as a binary variable (i.e. positive or negative), to determine the viability of alveolar echinococcosis, supposeably being less accurate than to Em18 [17]. Nonetheless, to the best of our knowledge, neither Em18 nor EgHF are currently used as quantitative parameters to evaluate the course of disease, probably also owing to the fact that the methods lack exact reproducibility in repeated measurements.

FDG-PET/CT is considered the “gold standard” for the evaluation of the viability of alveolar echinococcosis [15]. However, a negative PET/CT alone is also unreliable in identifying patients eligible for benzimidazole discontinuation, exposing patients to the risk of recurrence [7, 18]. Similar to the antibody levels, quantitative measures of metabolic activity in PET/CT are currently not used to evaluate the course of disease, neither in clinical routine nor experimentally in previous publications.

To date, double-negative results in PET/CT and Em18 serological tests are considered to represent the best marker for interruption of benzimidazole therapy in alveolar echinococcosis [16]. The relation of quantitative parameters of PET/CT and quantitative parameters of immunodiagnosis has not been studied previously. Our study shows, that the course of metabolic activity in echinococcosis manifestations in PET/CT in a patient correlates with the course of EgHF antibody levels. However, in some patients large discrepancies between trends of metabolic activity in PET/CT and EgHF antibody levels were documented, which may indicate, that the two identities are related, but may have to be evaluated independently, when treating patients with alveolar echinococcosis. Hence, we hypothesize, that quantitative parameters of PET/CT and Em18 serological tests may be used to assess the course of alveolar echinococcosis and potentially predict the duration of anthelmintic therapy.

### Limitations

In the present retrospective study, a rather small number of patients fulfilled the study inclusion criteria of at least two PET/CT examinations and an inoperable status of a rare disease. Furthermore, in only a few patients, benzimidazole therapy was successfully discontinued. Hence, statistical analyses of this endpoint had very limitated power, and further studies are required to confirm our findings. Finally, immunodiagnosis of only one antibody (i.e., EgHF) was continously performed in all patients during the study period of 17 years. Hence, similar analysis, especially concerning Em18 antibody levels, would be of interest in future studies.

## Conclusion

In inoperable patients with alveolar echinococcosis, the course of metabolic activity in follow-up PET/CT is associated to the course EgHF antibody levels. Both parameters may potentially be used to evaluate the course of the disease and potentially predict the duration of benzimidazole therapy.
